# Influence of Deposition Temperature on the Phase Evolution of HfNbTiVZr High-Entropy Thin Films

**DOI:** 10.3390/ma12040587

**Published:** 2019-02-15

**Authors:** Stefan Fritze, Christian M. Koller, Linus von Fieandt, Paulius Malinovskis, Kristina Johansson, Erik Lewin, Paul H. Mayrhofer, Ulf Jansson

**Affiliations:** 1 Department of Chemistry-Ångström, Uppsala University, SE-75120 Uppsala, Sweden; linus.fieandt@kemi.uu.se or linus.von_fieandt@sandvik.com (L.v.F); p.malinovskis@gmail.com (P.M.); Kristina.johansson@kemi.uu.se (K.J.); erik.lewin@kemi.uu.se (E.L.); ulf.jansson@kemi.uu.se (U.J.); 2 Institute of Materials Science and Technology, TU Wien, A-1060 Wien, Austria; christian.martin.koller@tuwien.ac.at or christian.koller@pankl.com (C.M.K.); paul.mayrhofer@tuwien.ac.at (P.H.M.)

**Keywords:** high-entropy alloys, physical vapour deposition (PVD), metallic glass

## Abstract

In this study, we show that the phase formation of HfNbTiVZr high-entropy thin films is strongly influenced by the substrate temperature. Films deposited at room temperature exhibit an amorphous microstructure and are 6.5 GPa hard. With increasing substrate temperature (room temperature to 275 °C), a transition from an amorphous to a single-phased body-centred cubic (bcc) solid solution occurs, resulting in a hardness increase to 7.9 GPa. A higher deposition temperature (450 °C) leads to the formation of C14 or C15 Laves phase precipitates in the bcc matrix and a further enhancement of mechanical properties with a peak hardness value of 9.2 GPa. These results also show that thin films follow different phase formation pathways compared to HfNbTiVZr bulk alloys.

## 1. Introduction

Recently, the concept of high-entropy alloys (HEAs) was introduced as a new approach to design materials [1,2]. In the initial HEA concept [2], five or more elements were mixed close to equimolar concentrations. This led to high-entropy mixing, which stabilised random solid solutions over the formation of intermetallic compounds. In the initial HEA concept [2], five or more elements are mixed in close to equimolar concentrations. This will lead to a high entropy of mixing, which stabilises random solid solutions over the formation of intermetallic compounds. Most HEAs have a simple bcc (body-centred cubic) structure or an fcc (face-centred cubic)-like structure. An example is HEAs based on refractory metals (early transition metals in groups 4–6), which typically crystallise in a bcc structure. These alloys are reported to exhibit interesting mechanical properties, especially at elevated temperatures, suggesting a promising pathway for new high-performance materials [3,4,5,6,7,8,9]. An example is HfNbTiVZr, which forms a single-phase bcc solid solution in the as-synthesised state and therefore has been described as an HEA [10,11]. Sahlberg et al. have recently demonstrated that this alloy has superior hydrogen storage properties, and therefore may have a potential use in future energy applications [12,13].

A potential problem with HEAs is the fact that they can transform into intermetallic phases, such as Laves phases, which often exhibit brittle behaviour [14,15]. However, recent studies have shown the positive influence of intermetallic phases on the mechanical properties of some HEAs [16,17]. Fazakas et al. have reported the formation of a cubic C15 Laves (with an AB_2_ structure and a r_A_/r_B_ ≈ 1.22) phase upon annealing to 900 °C [10]. The formation of Laves phases in this system is also supported by a recent study by Yurchenko et al. showing a correlation between Laves phase formation, lattice distortion, and electronegativity [18]. In a recent paper, we have demonstrated that bulk HfNbTiVZr indeed is a HEA with a bcc structure but only at high temperatures (>800 °C), where the contribution from the entropy of mixing is large enough to favour a single solid solution phase [19]. At lower temperatures, the CALPHAD calculations suggest that the thermodynamically stable phase composition is a mixture of a bcc phase, a hexagonal close packed (hcp) phase, and a cubic C15 Laves phase (MgCu_2_). Annealing experiments support the thermodynamic calculations but show that a hexagonal C14 Laves (MgZn_2_) is formed [19]. Whether the MgCu_2_ type or the MgZn_2_ type Laves phase forms, strongly depends on the Valence Electron Concentration (VEC), where lower VECs prefer fcc and VECs above 1.8 prefer hcp [20]. Furthermore, the results in reference [19] and several other papers on HEAs suggest that kinetic factors are important [21,22]. During solidification from the melt, the alloy is stable as a solid solution due to the high entropy of mixing. At lower temperatures, the alloy should transform to a multi-phase material by, e.g., precipitation reactions in the solid state. However, this requires a significant diffusion in the solid state. If the cooling rate is high enough, the alloy will remain in a solid solution phase and form a metastable multicomponent alloy. Additionally, metastable bulk metallic glasses can be formed from the melt if the cooling rate exceeds a critical value. These metastable alloys will decompose into intermetallic phases during annealing. 

Most studies on HEAs have been carried out on bulk samples synthesised from melt. Recently, magnetron sputtering of HEAs has been utilised as an alternative method to produce coatings with functional properties [23,24,25,26,27]. Magnetron sputtering from the gas phase leads to extremely high quenching rates (>10^6^ K/s [28]) of the adsorbed atoms during growth. This means that the impinging atoms on the surface will quickly lose energy and be incorporated into the growing film. This will favour simple, single-phase solutions with bcc and fcc like structures. The extremely high quenching rates during magnetron sputtering also enable the synthesis of amorphous metals, which cannot be synthesised as bulk samples. Consequently, we should expect that many alloys will appear to be HEAs or metallic glasses, although they should form a mixture of intermetallic phases from a thermodynamical point of view. The high quenching rate in magnetron sputtering may also lead to completely different phase compositions compared to conventional casting methods. 

The aim of the present study is to investigate the possibilities of depositing HfNbTiVZr thin films with magnetron sputtering and to study the phase composition of these films compared to the bulk samples in reference [19]. In particular, we will investigate if the phase composition of the deposited films is different from similar samples obtained by arc-melting.

## 2. Materials and Methods 

The HfNbTiVZr thin films were deposited by non-reactive DC-magnetron sputtering in an ultra-high vacuum chamber (base pressure of <5 × 10^−8^ Pa). The system was equipped with four 2″ magnetrons in a confocal sputter-down configuration. Elemental targets of Hf, Ti, and V were used on three of the magnetrons. The fourth magnetron was equipped with a segmented target, composed of one half Zr and one half Nb. An Ar^+^ plasma was ignited at 0.4 Pa, using a 42 sccm Ar gas flow. A detailed description of the experimental chamber can be found in reference [29,30]. Prior to the depositions, the single-crystal Si(001) and α-Al_2_O_3_(00l) substrates were pre-heated to the desired temperature for at least 60 min to minimise the temperature gradient, and the substrates were biased to −50 V. Three different temperatures were used: room temperature (RT), 275 °C, and 450 °C, where RT implies that no external heating was applied, i.e., the substrate was only heated by the arriving high-energetic sputtered particles. The film composition was analysed by X-ray photoelectron spectroscopy (XPS) using a PHI Quantum 2000 spectrometer (Physical Electronics, Eden Prairie, MN, USA) with monochromatic Al K_α_ radiation. The sensitivity factors were calibrated against a bulk equimolar HfNbTiVZr sample, and the binding energy scale was calibrated against reference samples of Au, Ag, and Cu. The chemical composition of the films was determined from depth profiles, acquired with 2 kV Ar^+^ ions. The structural properties were determined by X-ray diffraction (XRD) measurements, carried out using a Siemens D5000 diffractometer (Siemens, Munich, Germany) using a Cu Kα radiation source with parallel beam geometry. In addition, pole figures were measured between 0 and 89° in Ψ (tilt angle) using a Philips MRD-X’Pert diffractometer (Almelo, Overijssel, the Netherlands) operating in point focus mode and equipped with a primary X-ray poly-capillary lens with crossed slits, a secondary flat graphite monochromator with parallel plate collimator, and a nickel filter. The mechanical properties were determined on a CSM Instruments Ultra Nano Hardness Tester (Anton Paar GmbH, Peseux, Switzerland) equipped with a Berkovich diamond tip. The theoretical Poisson’s of ν = 0.388 was used to determine the Young’s modulus [31]. Transmission electron microscopy (TEM) investigations were carried out using a FEI Tecnai F20 TEM (200 kV, (Eindhoven, Nordbrabant, the Netherlands)) with an EDAX Apollo XLT2 energy-dispersive X-ray spectroscopy (EDS) detector for chemical analyses. Cross-sections were prepared by mechanical grinding and ion polishing. 

## 3. Results and Discussion

**Figure 1:**
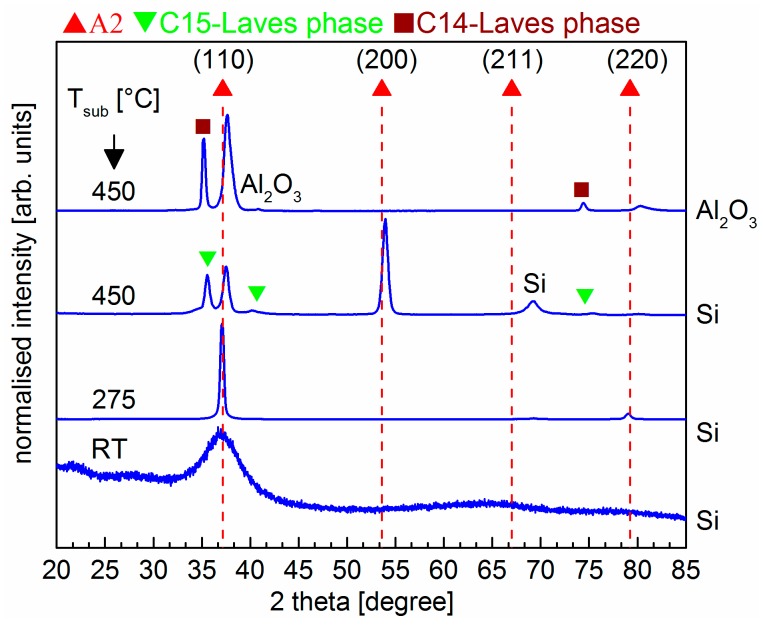
θ–2θ diffractograms of HfNbTiVZr thin films prepared with substrate temperature (T_sub_) = room temperature (RT), 275, and 450 °C. The T_sub_ values correspond to homologous temperature values of 0.17, 0.31, and 0.41. Red upward triangular markers with dashed vertical lines indicate the positions for a bcc phase with a lattice parameter of 3.42 Å.

XPS analysis showed that all coatings have near-equimolar Hf_21_Nb_20_Ti_20_V_18_Zr_21_ composition. Figure 1 shows the θ–2θ diffractograms of films deposited at the three different temperatures: RT, 275 °C, and 450 °C. A single diffuse, very broad peak is observed for the film deposited at RT in the XRD diffractogram, indicating an X-ray amorphous structure. The XRD pattern (Figure 1) shows that films deposited at a substrate temperature (T_sub_) of 275 °C crystallise in a simple bcc structure (A2, Im$\bar{3}$m) with a <110> preferred orientation without indications of additional phases. The lattice parameter was determined to be 3.42 Å, which is in good agreement with the value obtained by ab-initio calculations [10]. However, the lattice parameter was significantly larger than bulk Hf_20_Nb_20_Ti_20_V_20_Zr_20_ samples in reference [10], which can be attributed to the higher Hf and Zr contents (and lower V content) in the present coating. The XRD patterns also reveal (Figure 1) that films deposited at 450 °C contain secondary phases. The diffraction peaks of the bcc phase are shifted towards higher 2θ-values as result of a decrease in the cell parameter to 3.39 Å, which, for instance, can be attributed to a change in composition within the bcc phase. The observed I_110_/I_200_ peak ratio is changed due to a change from the preferred <110> growth orientation to a preferred <100> growth orientation. The change of preferred orientation can be a result of various factors, such as a higher substrate temperature and the competitive growth of two phases. The peaks at 35.5°, 40.3°, and 75.5° could be matched either with the peaks of a cubic C15 Laves phase, or the peaks of a hexagonal phase, such as the C14 Laves phase or hcp Zr. The results above were obtained with a Si substrate. During our work, we noticed that a sapphire Al_2_O_3_(00l) substrate resulted in a different texture and phase composition. This can be seen in the top diffractogram in Figure 1 acquired from a film deposited at 450 °C on a sapphire substrate. In this case, the bcc phase maintained the strong preferred <110> growth orientation and also the secondary phase exhibits a strong texture.

**Figure 2:**
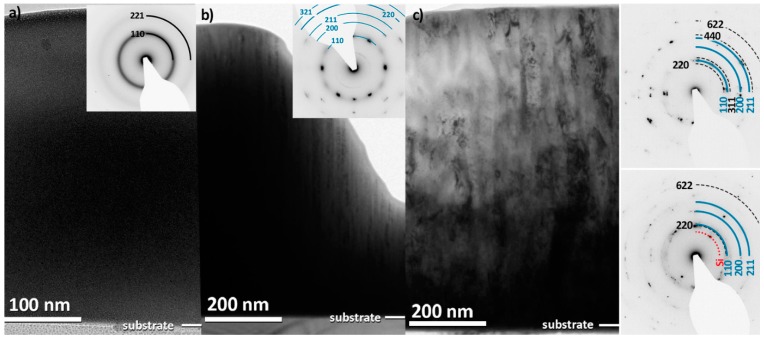
Transmission electron microscopy (TEM) cross-section of HfNbTiVZr films grown on Si substrates using a T_sub_ of (**a**) RT, (**b**) 275 °C, and (**c**) 450 °C. The respective selective area electron diffraction (SAED) patterns are inserted in the images where blue solid rings indicate the bcc phase (in (**b**) and (**c**)), and black dashed rings (in (**c**)) indicate the C15 Laves phase. The SAEDs presented in (**c**) were recorded with a distance of 200 nm and 600 nm from the Si substrate. The dotted red ring corresponds to the Si substrate.

The selective area electron diffraction pattern (SAED) of the film deposited at RT exhibits only broad (Figure 2a), featureless rings confirming the amorphous structure. This film will be referred to as metallic glass in the further text. The formation of an amorphous HfNbTiVZr phase is in contrast to casted alloys, which form well-crystallised bcc alloy with rather large grains (>50 µm) [13]. This can be attributed to the high quenching rate in magnetron sputtering combined with the low mobility of ad-atoms at low T_sub_, which together limit the ability to form crystalline phases. Furthermore, it is assumed that HEAs exhibit a reduced bulk diffusion rate of the constituents compared to conventional alloys, and this is often called the “sluggish diffusion” effect [32]. Such an effect can be present in HfNbTiVZr alloys and can lead to a further decrease in atomic mobility, which prevents the formation of a crystalline phase. 

**Figure 3:**
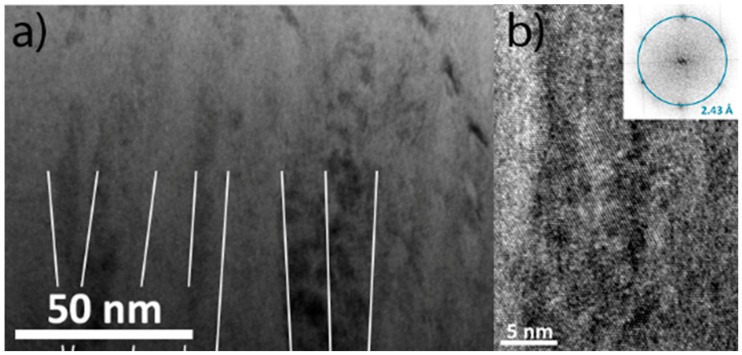
(**a**) TEM bright field (BF) image of the film deposited at 275 °C; (**b**) HR-TEM with Fast Fourier Transformation (FFT) inset.

The A2 structure of films deposited at 275 °C is confirmed by SAED. All diffraction spots in the pattern (Figure 2b) can be assigned to bcc reflections. The SAED pattern also reveals a <110> preferred orientation, consistent with the θ–2θ XRD measurements. The cross-sectional TEM bright field image in Figure 3a reveals a narrow columnar microstructure with column widths around 20 nm. The corresponding HR-TEM image in Figure 3b also reveals a dense structure with elongated grains, and the Fast Fourier Transformation (FFT) corroborates the bcc structure (see inset).

**Figure 4:**
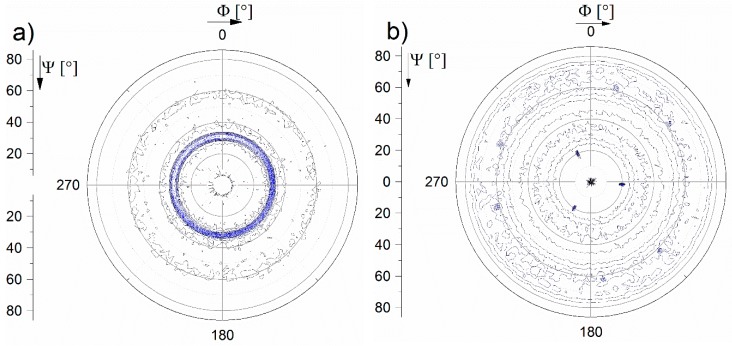
Pole figures obtained from films grown at T_sub_ = 450 °C on (**a**) a Si substrate using the (422) reflection from C15 Laves phase at 2θ = 64.4° (0–6° psi (tilt angle) were omitted due to strong reflection in the centre), and (**b**) an Al_2_O_3_ substrate using the (214) reflection from the C14 Laves phase (2θ = 68°).

A pole figure measurement of the film deposited at 450 °C on the Si substrate using the (422) peak (2θ = 64.4°) of the C15 Laves phase (MgCu_2_-type, Fd$\bar{3}$m) confirms that the secondary phase is a C15 Laves phase with a lattice parameter of 7.14 Å. The observed tilt angle of ~30° is a close match to the planar angle between the (422) and (220) planes of the C15 Laves phase (Figure 4a, blue ring). SAED carried out close to film/substrate interface (Figure 2c, bottom inset) shows diffraction spots on two separate rings. The d-spacings of these rings correspond to the bcc (110) and C15 Laves (220) peak and indicate that both phases are formed simultaneously. An SAED taken 600 nm from the substrate, shown in the insert in Figure 2c, corroborates the presence of both phases throughout the film.

In contrast, a pole figure measurement of the film deposited at 450 °C on the sapphire substrate using the (422) peak (2θ = 64.4°) of the C15 Laves phase revealed that the secondary phase is not cubic. Additional pole figure measurements using a 2θ = 68° showed that the secondary phase is hexagonal (Figure 4b). The peaks in the θ–2θ diffractogram can be fitted either to an hcp phase (A3, P63/mmc), with lattice parameters close to pure Zr [33], or to a C14 Laves phase (P63/mmc, MgZn_2_), with lattice parameters close to the phase in reference [19]. Peak overlaps of these two phases do not allow us to unambiguously separate between these two alternatives. However, the almost unchanged lattice parameter of the bcc phase suggests that the bcc phase is not solely depleted of elements with large atomic radii, such as Zr and Hf, and therefore the secondary phase is most likely a C14 Laves phase. The chemical composition of the C14 Laves phase is similar to the C15 Laves phase; the main difference is the AB stacking sequence instead of ABC [34]. This is also supported by the pole figure measurements, as the C14 laves phase gives the best fit (~22°, three sharp spots) from the (214) plane, whereas the A3 phase would result in a reflection in the pole figure at 26°. Furthermore, it can be concluded that the orientation of the C14 Laves phase is <103>.

Nanoindentation of the metallic glass film (see Table 1) shows a hardness of 6.5 ± 0.3 GPa and a Young’s modulus of 95 ± 3 GPa. The single-phase HEA film exhibits a hardness of 7.9 ± 0.3 GPa and a Young’s modulus of 105 ± 2 GPa. The increase of hardness is in agreement with the general trend that crystalline alloys are harder than the amorphous phase with identical composition [35]. The formation of C14 or C15 Laves phase precipitates leads to a further increase of hardness to 9.2 ± 0.4 GPa and a Young’s modulus of 118 ± 3 GPa. All HfNbTiVZr films exhibit a significantly higher hardness than the reported literature values for the respective bulk alloys of 3.8 GPa (observed for bcc and bcc + Laves phases) [10]. The higher hardness of the single-phase bcc film, compared to the bulk value, is attributed to the nanocrystalline microstructure resulting in grain refinement hardening [36].

The results above show similarities and clear differences between the bulk samples in reference [19] and magnetron sputtered HfNbTiVZr thin films. The bulk samples are prepared by a melting process, where the melt is solidified. The first phases formed in such a process are therefore often related to the high temperature phases given in the phase diagram. During cooling, phase transformations may occur in the solid state providing that the atomic diffusion is high enough. The thermodynamically most stable phase in the HfNbTiVZr system below 600 °C is a phase mixture of bcc, hcp, and a C15 Laves phase [19]. However, bulk samples prepared by arc-melting exhibit a single-phase solid solution bcc structure stable at high temperatures just below the melting point. The hcp and Laves phases are never observed in as-synthesised samples due to the rather high cooling rate. However, upon annealing both the hcp and a C14 Laves phases are formed above 600 °C [19]. 

It is clear that the magnetron sputtered films have a completely different phase composition and microstructure compared to the bulk samples. The high quenching rates of up to 10^6^ K/s combined with the low surface mobility (no substrate heating applied) enable the possibility to synthesise an amorphous HfNbTiVZr thin film, which is not accessible by conventional bulk synthesis techniques. Bulk samples and thin films prepared at T_sub_ = 275 °C both crystallise in a simple bcc structure. The crystalline film exhibits a very strong <110> texture. The main difference between the bulk alloy and the thin film is observed at 450 °C. On Si substrates, the film consists of a less textured bcc phase and a cubic C15 Laves phase. This is in strong contrast to the annealed bulk samples, which show no phase transformations at 450 °C, but above 600 °C an incoherent hexagonal C14 Laves phase and a hcp phase form. The texture and phase composition also depends on the substrate, as films deposited on Al_2_O_3_(00l) form a C14 Laves phase similar to the bulk samples after annealing above 600 °C.

The formation of Laves phases in the thin films, at much lower temperatures than during annealing of bulk samples, can be explained by the fact that surface diffusion (relevant during growth of the film) is typically much faster than bulk diffusion. The mobility of ad-atoms is much higher than within the bulk (of the same type of atoms) at the same temperature, and therefore any segregation or formation of a multi-phase structure would occur at lower temperatures during thin film growth than during annealing of a bulk sample. The prediction of C15 Laves phase as the most stable Laves phase in the HfNbTiVZr alloy is confirmed by calculations using a data base based on binaries. This means that the effect of, e.g., Nb on the Laves phase stability is not included [19]. It is known that Nb influences the stability of C14 versus C15 and that a single-phase area exists for some Nb contents in the Hf-Nb-V system [37]. Consequently, depending on the diffusivities and the availability of Nb can be different during film growth compared to a solid state transformation process. Further studies, however, are required to determine why the substrate can affect the C14/C15 formation

## 4. Conclusions

In summary, we have for the first time deposited near-equimolar HfNbTiVZr HEA thin films by DC-magnetron sputtering. The microstructure shows a strong dependence on the T_sub_, enabling the possibility of growing amorphous, single-phased bcc, or dual-phased thin films with different mechanical properties. The PVD process therefore allows for a “phase selection”, without post-treatments as typically needed for bulk HfNbTiVZr materials. Furthermore, the films deposited at higher temperatures form a two-phase microstructure of bcc HEA and C14 or C15 Laves phase depending on the substrate.

## Figures and Tables

**Table 1:** Deposition parameters phase composition, hardness H, and Young’s modulus E.

T_sub_ (°C)	Phases	H (GPa)	E (GPa)
RT	single-phase amorphous	6.5 ± 0.3	95 ± 3
275	single-phase bcc	7.9 ± 0.3	105 ± 2
450 *	bcc + Laves phases	9.2 ± 0.4	118 ± 3

* The hardness of 9.2 ± 0.4 is measured for the coatings containing a C14 Laves phase (on a Si substrate) and C15 Laves phase (on an Al_2_O_3_ substrate).
